# A Review of Coformer Utilization in Multicomponent Crystal Formation

**DOI:** 10.3390/molecules27248693

**Published:** 2022-12-08

**Authors:** Nasrul Wathoni, Wuri Ariestika Sari, Khaled M. Elamin, Ahmed Fouad Abdelwahab Mohammed, Ine Suharyani

**Affiliations:** 1 Department of Pharmaceutics and Pharmaceutical Technology, Faculty of Pharmacy, Universitas Padjadjaran, Sumedang 45363, Indonesia; 2 Research Center of Biopolymer for Drug and Cosmetic Delivery, Faculty of Pharmacy, Universitas Padjadjaran, Sumedang 45363, Indonesia; 3 Graduate School of Pharmaceutical Sciences, Kumamoto University, Kumamoto 862-0973, Japan; 4 Department of Pharmaceutics, Faculty of pharmacy, Minia University, Minia 61519, Egypt; 5 Sekolah Tinggi Farmasi Muhammadiyah Cirebon, Jl. Cideng Indah No.3, Cirebon 45153, Indonesia

**Keywords:** solubility enhancement, conformer, multicomponent crystal

## Abstract

Most recently discovered active pharmaceutical molecules and market-approved medicines are poorly soluble in water, resulting in limited drug bioavailability and therapeutic effectiveness. The application of coformers in a multicomponent crystal method is one possible strategy to modulate a drug’s solubility. A multicomponent crystal is a solid phase formed when several molecules of different substances crystallize in a crystal lattice with a certain stoichiometric ratio. The goal of this review paper is to comprehensively describe the application of coformers in the formation of multicomponent crystals as solutions for pharmaceutically active ingredients with limited solubility. Owing to their benefits including improved physicochemical profile of pharmaceutically active ingredients, multicomponent crystal methods are predicted to become increasingly prevalent in the development of active drug ingredients in the future

## 1. Introduction

Solubility is the phenomenon of dissolving a solute in a solvent to form a homogeneous system. Solubility is critical to achieving the desired drug concentration in systemic circulation to provide desired pharmacological effects. When a drug is administered orally, it must first dissolve in the gastrointestinal fluids before it can be absorbed into the bloodstream and reach the site of action [1,2].

The newly discovered drugs with low solubility and a high permeability (about 60% to 70%) belong to class II BCS (biopharmaceutical classification system), and those with a low solubility profile and low permeability are part of class IV BCS [3,4]. When administered orally, low-solubility drugs require higher doses to reach therapeutic plasma concentrations, which can cause problems associated with the drug delivery system, bioavailability, and therapeutic effectiveness [2]. The new compounds that were found in *Pinus armandii* Franch, namely 4′-methoxy-2-hydroxystilbene, 7,8-dihydro-2,4 (1h,3h)-pteridinedione, and 9h-pyrido [3,4-b] indole,7-methoxy-1,9-dimethyl-, among others, have potential to be used as biological substances in the pharmaceutical industry or as drugs, owing to their anti-inflammatory, anticancer, and anti-HIV activities [5]. Further research is needed to evaluate the role of these compounds as either active ingredients or coformers.

**Figure 1:**
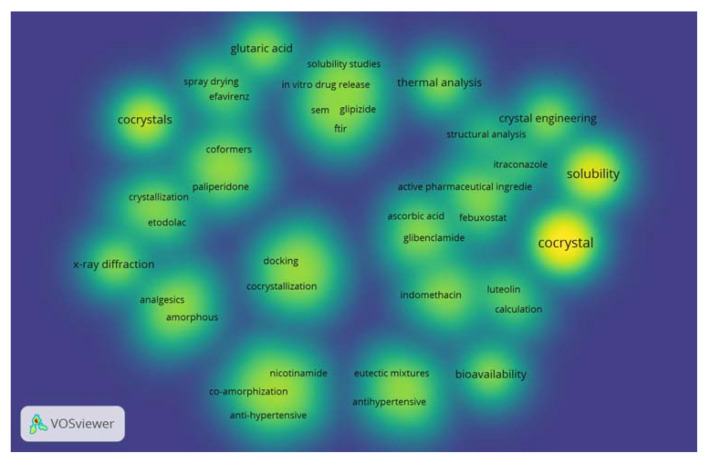
The density of coformer research in last 5 years.

The density analysis of coformer research conducted using VOSviewer software showed that many of studies have focused on improving the solubility of active pharmaceutical ingredients (Figure 1). The intensity of yellow color corresponds with the quantity of research.

**Figure 2:**
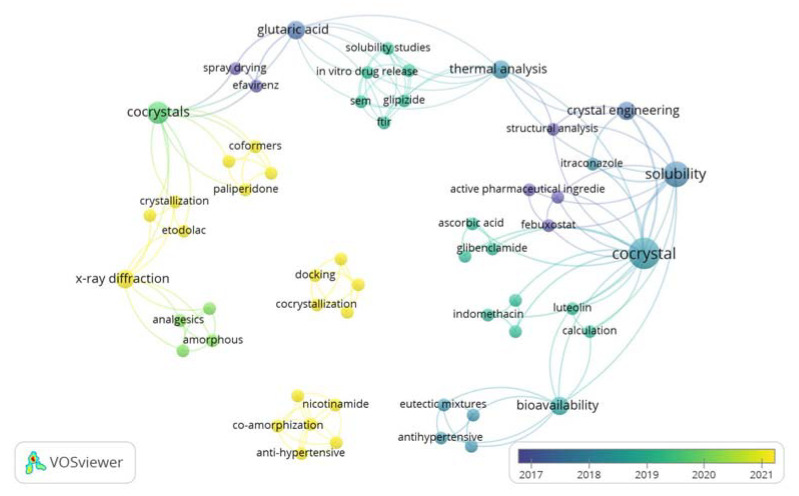
Analysis of coformer research in last 5 years using VOSviewer.

Many studies have been conducted with respect to the development of ways to modify the physicochemical properties of active pharmaceutical ingredients to increase their therapeutic effectiveness. Further analysis was conducted using VOSviewer to visualize the novelty of coformer research (Figure 2). Current research on coformers includes active substances such as glibenclamide, itraconazole, etodolac, febuxostat, gliplizide, efavirenz, indomethacin, etc. Ascorbic acid, glutaric acid, paliperidone, etc., have also been investigated as coformers.

One strategy to alter the solubility of a drug is to modify the crystal structure by combining the active pharmaceutical ingredient with coformers to form a crystalline multicomponent system, such as cocrystals and salts [3,4].

Multicomponent crystals are solid phases that form when more than one molecule of different substances crystallize together in a crystal lattice with a certain stoichiometric ratio. In salt-form drugs, the components are arranged in a crystal lattice based on their ion pairs, whereas in cocrystals, the components are arranged through weak interactions, such as hydrogen bonds, −π arrangement, or van der Waals interactions [6].

Coformers are cocrystallizing agents that interact non-ionically with active pharmaceutical ingredients in a crystal lattice; they have high water solubility and are usually non-volatile. Active ingredients and suitable coformers cause interactions through hydrogen bonds, van der Waals interaction, and -π stacking [7]. The selection of coformers is a significant obstacle in the formation of multicomponent pharmaceutical crystals suited for specific active medicinal components. The ability of the coformer to create hydrogen bonds with the medicinal molecule is usually used to select a coformer. Coformers also need to be GRAS (generally recognized as safe) in order to confirm their non-toxicity and safety [8].

The aim of this review article is to systematically describe the use of coformers in the formation of multicomponent crystals as solutions for pharmaceutically active substances with limited solubility. The main obstacles to developing new drug products are poor water solubility and low oral bioavailability of the active substance. Thus, the use of multicomponent crystals is an approach that can be used to increase the solubility of poorly water-soluble drugs and help improve physicochemical properties of pharmaceutically substances, such as melting point, tabletability, stability, bioavailability, and permeability.

## 2. Methods

**Figure 3:**
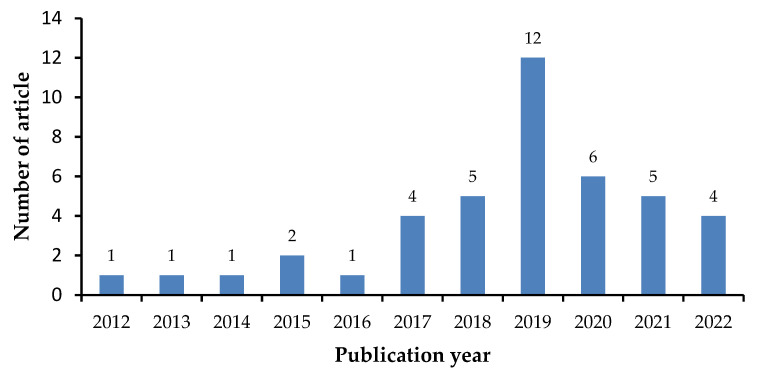
Number of articles per year.

**Figure 4:**
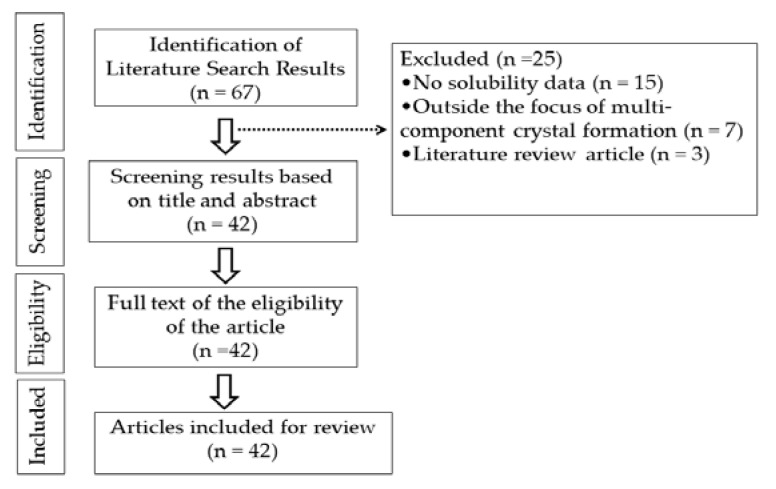
Data extraction.

The present study was conducted as literature search; articles were collected from Google Scholar, Pubmed, and Scopus using the keywords coformer, multicomponent crystal, cocrystal, salt, and solubility. Libraries were selected based on inclusion and exclusion criteria. Inclusion criteria were literature that contains information on the usage of coformers in the formation of multicomponent crystals for pharmaceutically active ingredients with limited solubility and a publication year of 2012–2022 (Figure 3). Review papers, publications not available as full text, and research involving methods other than the creation of multicomponent crystals were excluded (Figure 4).

## 3. Coformer Selection

The selection of coformers is a major challenge the development of pharmaceutical multicomponent crystals that are compatible with pharmaceutically active substances. The general strategy used in selecting coformers is tactless experimental approaches whereby a predetermined number of candidates from compounds listed in the generally regarded as safe (GRAS) list are used to form multicomponent crystals [9]. The requirements for a substance to be used as a coformer are that it must be safe and non-toxic and have no side effects. Coformers must be on the FDA’s “Everything added to food in the United States” (EAFUS) list or on the generally regarded as safe (GRAS) list. [10].

Several approaches can be adopted to select coformers, including the molecular synthon approach using the Cambridge Structural Database (CSD). The CSD provides information about the molecular associations of drugs and coformers based on functional groups bound to supramolecular synthons. A suitable coformer library can be prepared by CSD for the API. CSD is a computer-based approach used to find pairs of coformers and active pharmaceutical ingredients to form multicomponent crystals, as well as reduce costs and experimental time to increase research efficiency [7,11].

pKa is a simple approximation used to predict the formation of multicomponent crystals. In general, salt formation occurs when there is a difference between the pKa of a base and the pKa of an acid (ΔpKa) > 3, whereas pKa < 0 results in the formation of cocrystals. The value of pKa and the probability of proton transfer between acid–base pairs was found to show a linear relationship. Cruz-Cabeza et al. analyzed the differences in pKa (ΔpKa) values for 6465 crystalline complexes containing acid–base pairs and separated the complexes into three zones based on pKa values. In zone 1 (ΔpKa < −1), approximately 99.1% of crystal complexes were observed to be non-ionized, i.e., no proton transfer, whereas, in zone 3 (ΔpKa > 4), 99.2% of crystal complexes were observed as both ionized and non-ionized crystal structures, indicating that ionization occurs, namely the complete transfer of protons. However, in zone 2 (−1 pKa ≥ 4), the ionized complex increased linearly with ab increasing pKa value [12].

The Hansen solubility parameter is another important approach used to measure drug and coformer miscibility for crystallization under a multicomponent approach. According to the Hansen solubility parameter, the total cohesive energy of the liquid is divided into contributions from atomic dispersion (δd), dipole–dipole/polar interactions (δp), and hydrogen bonding (δh). Cohesive energy parameters can predict physicochemical properties such as melting point and solubility of a compound. Cohesive energy is the sum of van der Waals bonding forces, covalent bonds, hydrogen bonds, and ionic bonds. Cohesive energy is the energy required to break all these interactions and allow atoms or molecules to detach, resulting in the transformation of solids to liquid/gas or liquids to gas. If the cutoff value of the active compound and the coformer is less than 7 MPa^0.5^, it is considered miscible and capable of forming cocrystals [1,13]. Cocrystals can be formed depending on the cutoff value and radius of the active compound and the conformer [13].

In the selection of coformers, functional groups are needed to form molecular synthon bonds to predict the formation of cocrystals. A molecular synthon is a structural unit within a supermolecule that can be formed through known or imagined intermolecular interactions. Supramolecular synthons are divided into two classes, namely supramolecular heterosynthones and supramolecular homosynthones. Supramolecular heterosynthone is formed by covalent binding of different molecular fungtional groups but complements, for example, carboxylic acid–pyridine and carboxylic–amide. Homosynthone is produced by the formation of the same dimer formation C=O..H-N hydrogen binding [11].

For example, the interaction between the carboxylic acid group and the amide is occurs in two or more compounds with different but complementary functions. Meanwhile, supramolecular homosynthone is a formed between two similar compounds with the same functional group as the interaction between an amide group and an amide or a carboxylic acid group with a carboxylic acid [11].

In a study conducted by Prasad et al., cocrystal formation in an ornidazole system with para-amino acid and para-hydroxybenzoic acid coformers was analyzed as follows. For a binary system with strong hydrogen-bonding groups, the primary supramolecular growth unit must be at least three molecules long to form a cocrystal. The strong hydrogen bonding groups in ornidazole are imidazole and nitro groups, which generally form imidazole–carboxylic acid and nitro-amine/hydroxyl/iodo heterosynthone, giving rise to cocrystals. All benzoic acid coformers can form carboxylic acid–imidazole heterodimeric units when combined with ornidazole. However, cocrystal formation depends on the strength of the complementary induction between the para-functionality of the coformer and the nitro group of ornidazole, and the combination can propagate as cocrystal growth units. The coformers of PABA and PHBA with ornidazole each form a strong amine/hydroxyl–nitro interaction according to the complementary induction strength between the functional groups, generating a supramolecular unit beyond the imidazole–acid heterodimer with ORL and therefore producing a cocrystal [14].

## 4. Multicomponent Crystal Formation

**Figure 5:**
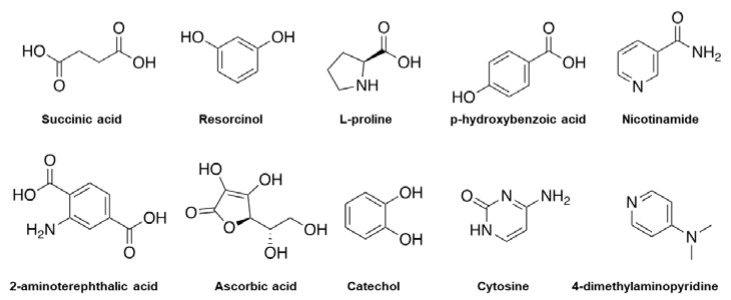
Examples of chemical molecules used for coformer formation.

Multicomponent Crystal formed by different ways such as solvent evaporation, slurry conversion, Cooling cocrystallization, neat grinding, Liquid-assisted grinding, and spray drying (Table 1), (Figure 5). Each method was chosen depending on the characteristics both of the drug and coformer. 

**Figure 6:**
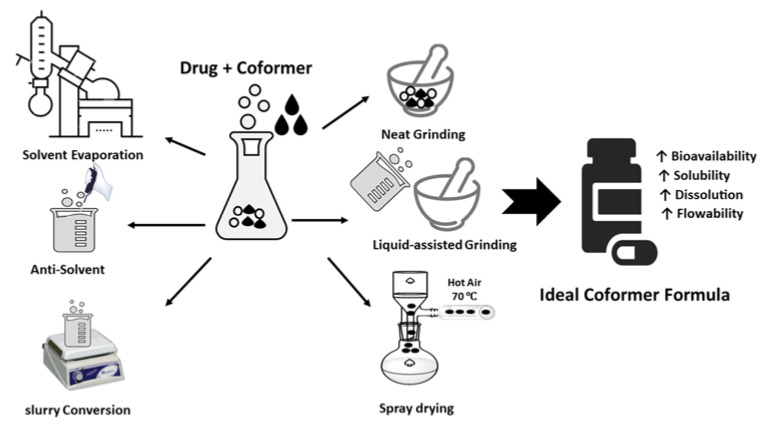
Coformer synthesis techniques.

Methods of forming multicomponent crystals can be classified as either thermodynamic or kinetic methods (Figure 6). Kinetic method deals with non-equilibrium conditions depending on the energy of the system and the duration of the reaction. These methods are derived primarily from metastable cocrystal forms, which have higher Gibbs free energies than stable forms. Examples of kinetically based multicomponent crystal formation methods include grinding technology, spray drying, slurry sonication, and supercritical fluid technology. Using the thermodynamic method, the reaction occurs under equilibrium conditions and usually takes a long time. An example is the solvent evaporation method. Another classification of multicomponent crystal formation methods relates to the use of solvents. Multicomponent crystal formation techniques are also classified as solvent-based methods or solvent-free methods [57].

### 4.1. Solvent Evaporation

Solvent evaporation is the most commonly used method for laboratory-scale multicomponent crystal formation, during which the drug and coformer are dissolved at a given stoichiometric ratio in a suitable solvent. Stirring is carried out under constant conditions to facilitate molecular interactions between the drug and the conformer, and then the solvent is allowed to evaporate, forming a solid, namely crystals. The main requirement of this method is that the components in forming multicomponent crystals must be completely soluble in the solvent [58].

This is a simple and effective method to identify the optimal combination of active pharmaceutical ingredients, coformers, and solvents. However, the solvent evaporation approach is subject to several limitations. For example, when the co-crystallization process occurs between two components that are not aligned, the component with lower solubility precipitates, forming a solid mixture of cocrystal components, and cocrystals may fail to form. In addition, unwanted solvents may form [57,59]. To avoid this, the construction of a phase diagram can help to determine the pure cocrystal region. 

The solvent evaporation technique has been successfully used to form multicomponent crystals and has succeeded in increasing the solubility of pharmaceutically active substances [15,16,17,18,19,20,21,22,23,24,28].

### 4.2. Antisolvent

This method utilizes a continuous co-crystallization process to optimize the quality, particle size, and cocrystal characteristics. The addition of an antisolvent reduces the solubility of the cocrystal until it reaches a supersaturated condition, resulting in cocrystal precipitation. As a result, the choice of solvent combination is critical, as the cocrystal must have poor solubility when little solvent is used. Solvent/antisolvent mixtures used to generate cocrystals include ethanol/water, ethanol/acetonitrile, and ethanol/ethyl acetate [60,61].

This method represents an alternative to solvent evaporation and cooling techniques for the formation of cocrystals with lower solubility. In addition, the process can be performed at ambient temperature and consumes less energy than cooling and solvent evaporation methods. Several studies have investigated techniques involving antisolvents to make it easier to control the properties of crystals and increase their purity and yield [46].

### 4.3. Cooling Co-Crystallization

Cooling co-crystallization is based on temperature variations and generally uses a reactor to mix components and solvents. The system is then heated to achieve dissolution of the two components. Saturation is achieved by reducing the temperature. This approach can be used with a complementary phase diagram to identify thermodynamically stable regions and predict potential cocrystal formation. In addition, by analyzing the kinetic pathways of the compounds and the degree of saturation, it is possible to determine the optimal conditions [57].

Research by Ahhirao et al. using the cooling crystallization method for the preparation of etodolac–glutamic acid cocrystals showed a significant increase in solubility and dissolution rate compared to the parent compound. Etodolac was dissolved in methanol, and glutamic acid was dissolved in distilled water. The drug solution was added to the coformer solution. The resulting mixture was kept in the refrigerator overnight and filtered to obtain cocrystals. The solution was then filtered to remove the insoluble materials [32].

### 4.4. Slurry Conversion

This method involves the formation of cocrystals by adding excess cocrystal components to the solvent. In this method, each component dissolves slowly to form a complex, which may result in the formation of cocrystals. Slurry conversion is an alternative method of producing cocrystals, the coformers of which are insufficiently soluble. The slurry conversion method is simple and includes the crystallization of pure cocrystals with a small quantity of solvent. This approach can be used to produce cocrystals only if the cocrystal is the most thermodynamically stable form relative to other crystalline forms. As a result, this approach can also be used to screen for the most stable crystal formations [62].

According this method, a coformer and drug are combined in equimolar amounts and slurried at 250 rpm overnight. The solution is then allowed to evaporate slowly for 48 h at room temperature. The resulting solid crystal is filtered, washed, dried, sieved, and stored for further evaluation [63].

### 4.5. Neat Grinding

Neat grinding, also called dry grinding or solid grinding, consists of mixing the stoichiometric cocrystal components together in a solid state and grinding them either manually using a mortar and pestle or mechanically using a ball mill or vibratory mill. Manual net grinding is associated with some reproducibility problems, so the mechanical method is more efficient. Mefenamic acid cocrystals with nicotinamide coformers, itraconazole co-crystals with succinic acid coformers, aspartic acid, serine, proline, and glycine were obtained by this technique [15,40].

### 4.6. Liquid-Assisted Grinding

Liquid-assisted grinding involves the addition of a small amount of solvent to a dry solid prior to the start of the grinding process. The solvent acts as a catalyst, assisting in the formation of multicomponent crystals and must persist throughout the milling process. Liquid-assisted grinding results in more efficient multicomponent crystal formation and an increase in cocrystal formation kinetics compared to neat grinding [57].

In this process, the solvent acts as a catalyst, either as a medium that facilitates molecular diffusion or as an important factor in the formation of a multicomponent inclusion framework, and is therefore used to increase supramolecular selectivity in crystal systems. The solvent effect can be described as catalytic, as the low catalyst content is not part of the final product [64].

### 4.7. Spray Drying

Spray drying is a rapid method used to engineer a solid state, producing a dry powder from a solution or suspension using a stream of hot air. For coformer-pharmaceutically active substance solubility systems, in pure crystalline multicomponents cannot be formed using the solvent evaporation method, the spray-drying method can be used as an alternative. Carbamazepine–glutamic acid, theophylline–nicotinamide, urea succinic acid, and caffeine–glutamic acid cocrystals are examples of incongruent systems that cannot produce pure cocrystals by the solvent evaporation method but manage to form pure crystals when the spray drying-method is used [65].

Co-crystallization using the spray drying method was also reported by Patil et al. using carbamazepine and nicotinamide as coformer drugs and models. In brief, the carbamazepine–nicotinamide cocrystals produced by the spray drying-method have similar characteristics to the cocrystals produced by the liquid-assisted grinding method [56].

## 5. Multicomponent Crystal Development Strategy

**Figure 7:**
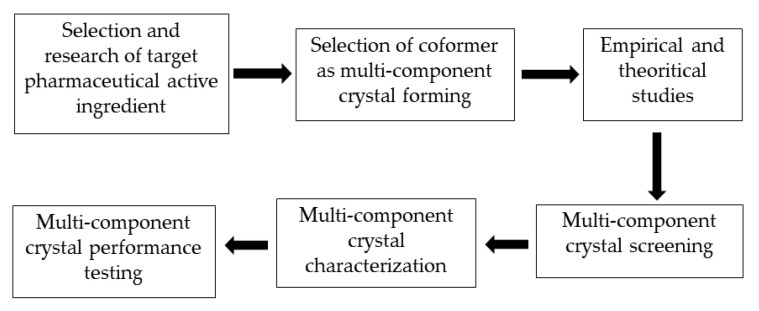
Stages of a multicomponent crystal development strategy.

The stages of a multicomponent crystal development strategy required to increase the solubility of a pharmaceutically active ingredient are presented in Figure 7. The first step in the development of a multicomponent crystal that sis compatible with a particular active substance is to study the molecular structure of the pharmaceutically active substance and identify functional groups that can form intermolecular interactions with appropriate coformers, such as van der Waals bonds, -π stacking, and hydrogen bond interactions. The next step is to select a multicomponent crystal-forming method, namely coformers.

The main requirement for a coformer is that it must be safe and not cause side effects. Coformers must be registered as generally regarded as safe (GRAS), indicating their safety and non-toxicity. Coformer selection is an important step in the design of multicomponent crystals [7,11].

During the coformer selection process in the design of multicomponent crystals, empirical and theoretical reference sources can be utilized, such as the Cambridge Structural Database (CSD) approach, Hansen’s solubility parameters, hydrogen bond theory, and pKa values. By using this reference source and approach, we are hopeful that suitable multicomponent crystal-forming pairs can be identified, and experimental costs and research time can be reduced. According to previous research, certain functional groups, such as alcohols, carboxylic acids, and amides, are suitable for the formation of supramolecular heterosynthone [66].

Multicomponent crystal screening is an experimental process used to determine whether a particular candidate coformer is capable of crystallizing with the targeted pharmaceutically active substance. After small-scale screening, the appropriate coformer can be selected. Various screening methods have been developed for the screening of multicomponent crystals. The solution method is useful for screening, whereby a small amount of a crystalline multicomponent in a given stoichiometric ratio is dissolved in a solvent, and a product is obtained by evaporation for analysis [67].

The next stage is multicomponent crystal characterization, with the aim of determining the crystal’s physical, chemical, and crystallographic properties. Generally, characterization includes chemical structural conformation and crystallographic analysis of the newly formed supramolecular synthone, as week as its thermal features, stability, and solubility. The final step of cocrystal design and preparation is performance testing of the newly formed compound, which includes in vitro and in vivo experiments. In vitro assays focus on intrinsic dissolution and dissolution assays, whereas in vivo assays concern the measurement of animal bioavailability and the rate and extent of pharmaceutically active substances reaching systemic circulation [67].

## 6. Authors’ Perspective

The formation of multicomponent crystals is a promising alternative solution in drug development to increase the solubility, bioavailability, and stability of drugs. Screening of coformers and the selection of multicomponent crystal formation techniques can lead to the development of suitable multicomponent crystals. The selection of coformers is a major challenge in the development of multicomponent pharmaceutical crystals that are compatible with certain pharmaceutically active substances. In the initial screening of coformers, the Cambridge Structural Database (CSD) approach, hydrogen bond theory, pKa values, and Hansen solubility parameters can be used. By using this reference source and approach, we are hopeful that suitable multicomponent crystal-forming pairs can be identified, and experimental costs and research time can be reduced.

There are also some factors that can affect the success of the formation of multicomponent crystals, including the number of donor and hydrogen bond acceptor groups in pharmaceutically active substances and coformers. The flexibility of functional groups also plays a role in determining the success of multicomponent crystals.

## 7. Conclusions

In this review article, various technologies used for experimental screening for the manufacture of pharmaceutical crystal multicomponents are presented as solutions to overcome the poor solubility profile of pharmaceutically active substances. In the manufacture of multicomponent crystals, several techniques can be applied both on a laboratory scale and on a larger scale. The technique used to form multicomponent crystals can be adjusted based on the properties of the pharmaceutically active substance, the selected coformer, and technological capabilities. We anticipate that the multicomponent crystal approach will become increasingly common in the development of pharmaceutically active substances in the future, owing to its continued benefits, such as improved physicochemical profile of pharmaceutically active ingredients. Thus, coformer formation can be applied to improve both the solubility and permeability of active susbtances, resulting in increased bioavailability.

## Figures and Tables

**Table 1:** Solubility data of multicomponent crystals of active pharmaceutical ingredients with coformers.

Method	Active Pharmaceutical Ingredient	Coformer	Solubility Enhancement (Fold)	Reference
Solvent evaporation	Atorvastatin kalsium	Succinic acid	1.5	[15]
	Acetazolamide	Proline	3.57	[16]
	Metaxalone	Nicotinamide	8.6	[17]
		Salicylamide	4.5	
		4-hydroxybenzoic acid	5	
	Norfloxacin	Resorcinol	10	[18]
	Furosemide	5-fluorocytosine	2.7	[19]
	Candesartan	Methyl paraben	6.94	[20]
	Naringenin	L-proline	2	[21]
		Betaine	3.35	
	Xanthohumol	Acetamide	2.6	[22]
		Glutarimide	1.6	
		Nicotinamide	1.4	
		Caffeine	1.3	
	Paliperidone	Benzamide	61	[23]
		Boric acid	1.48	
		Nicotinamide	18.5	
		p-hydroxy benzoic acid (PHBA)	141	
	Glibenclamide	Ascorbic acid	26	[24]
	Ibuprofen	Nicotinamide	70	[25]
	Diclofenac potassium	L-Proline	3.56	[26]
	Hesperidin	Piperine	1.9	[27]
	Etil p-methoxycinnamate	Citric acid	1.4	[28]
	Itraconazole	Trans-cinnamic acid	1.97	[29]
		Sebacic acid	1.09	
Slurry conversion	Telmisartan	Gentisic acid	3.7	[30]
		Maleic acid	4.4	
		Para-aminobenzoic acid	5	
		Adipic acid	5.4	
	Valsartan	Nicotinamide	2.19	[31]
Cooling cocrystallization	Etodolac	Glutaric acid	3.61	[32]
Neat grinding	Mefenamic acid	Nicotinamide	2.56	[33]
	Zoledronic acid	Nicotinamide	20.12	[34]
	Itraconazole	Aspartate acid	3.1	[35]
		Proline	2.1	
		Serine	2.5	
		Glycine	2.3	
		Succinic acid	1.62	
Liquid-assisted grinding	Hydrochlorothiazide	Nicotinic acid	2	[36]
		4-aminobenzoic acid	2.4	
		Nicotinamide	1.3	
	Olanzapine	2-aminoterephthalic acid	5	[37]
	Daidzein	Isocotinamid	2.1	[38]
		Cytosine	1.9	
		Theobromine	1.7	
	Irbesartan	Ascorbic acid	7.3	[39]
		Nicotinc acid	7	
		Syringic acid	4.4	
	Ethenzamid	Maleic acid	1.8	[40]
	Ritonavir	L-tyrosine	11.24	[41]
	Oxyresveratol	Citric acid	1.3	[42]
	Felodipine	Imidazole	2	[43]
	Febuxostat	L-pyroglutamic acid	4	[44]
	Diacerein	2,4-dihydroxybenzoic acid	2	[45]
	Carbamazepine	Naphthoic acid	2	[46]
	Hydrochlorothiazide	4- dimethylaminopyridine	4	[47]
		Picolinamide	0.5	
		Phenazine	1.4	
	Sildenafil	Glutamate	3.2	[48]
		Pimelic acid	1.3	
	Gliclazide	Piperazine	6.6	[49]
		Catechol	6	
		Resorcinol	3.5	
		p-toluenesulfonic acid	2.6	
	Glibenclamide	Succinic acid	3.5	[50]
		Nicotinic acid	3	
		Hippuric acid	2.2	
		Theophylline	1.5	
	Furosemide	Cytosine	11	[51]
		Adenine	7	
		Caffeine	6	
	Luteolin	Isoniazid	3.2	[52]
		Caffeine	2.1	
Spray drying	Efavirenz	Glutaric acid	2	[53]
	Plumbagin	Nicotinamide	2	[54]
	Furosemide	L-arginine	24	[55]
	Carbamazepine	Nicotinamide	4.1	[56]

## Data Availability

Not applicable.
